# Delayed Phenotypic Expression of Growth Hormone Transgenesis during Early Ontogeny in Atlantic Salmon (*Salmo salar*)?

**DOI:** 10.1371/journal.pone.0095853

**Published:** 2014-04-24

**Authors:** Darek T. R. Moreau, A. Kurt Gamperl, Garth L. Fletcher, Ian A. Fleming

**Affiliations:** 1 Department of Ocean Sciences and Cognitive and Behavioural Ecology Programme, Memorial University of Newfoundland, St. John's, Newfoundland & Labrador, Canada; 2 Department of Ocean Sciences, Memorial University of Newfoundland, St. John's, Newfoundland & Labrador, Canada; National University of Singapore, Singapore

## Abstract

Should growth hormone (GH) transgenic Atlantic salmon escape, there may be the potential for ecological and genetic impacts on wild populations. This study compared the developmental rate and respiratory metabolism of GH transgenic and non-transgenic full sibling Atlantic salmon during early ontogeny; a life history period of intense selection that may provide critical insight into the fitness consequences of escaped transgenics. Transgenesis did not affect the routine oxygen consumption of eyed embryos, newly hatched larvae or first-feeding juveniles. Moreover, the timing of early life history events was similar, with transgenic fish hatching less than one day earlier, on average, than their non-transgenic siblings. As the start of exogenous feeding neared, however, transgenic fish were somewhat developmentally behind, having more unused yolk and being slightly smaller than their non-transgenic siblings. Although such differences were found between transgenic and non-transgenic siblings, family differences were more important in explaining phenotypic variation. These findings suggest that biologically significant differences in fitness-related traits between GH transgenic and non-transgenic Atlantic salmon were less than family differences during the earliest life stages. The implications of these results are discussed in light of the ecological risk assessment of genetically modified animals.

## Introduction

There is considerable interest in the application of transgenic biotechnologies to enhance animal production. Among the first animal biotechnologies to be considered commercially are growth hormone (GH) transgenic Atlantic salmon (*Salmo salar* L.). Similar to conventional aquaculture [1]–[3], there are concerns regarding the potential impacts of ecological and genetic interactions between transgenic and wild fish in nature [4]–[6]. As such, there is a need for empirical data with which to assess the possible environmental risks of such transgenic fish.

Early ontogeny is a period of intense selection in many fish species, and thus, may provide critical information regarding the fitness of transgenic fish strains relative to wild-type individuals. For example, salmon eggs incubate in buried gravel nests that can experience lethally low levels of dissolved oxygen, resulting in high mortality [7]–[9]. Upon hatch, alevins (larval phase) remain underneath the gravel until their endogenous yolk reserves are near fully consumed. At this point, individuals emerge and commence exogenous feeding. First-feeding is a critical period of survival and performance for many fish species, including salmon, where the fry (early stage juveniles) must learn to attain food, compete for and/or migrate to foraging territories, and avoid predation [10]–[12]. Mortality during the first few weeks of life can be greater than 80% [13]–[15]. Thus, any transgene-induced effects on physiological and behavioural traits during early ontogeny may impact the persistence of the transgene in nature.

Beyond its effects on growth [16], [17], GH transgensis is known to have pleiotropic effects on other phenotypic traits in salmon, including elevated metabolic rates, increased foraging motivation and reduced anti-predator behaviour [18]–[23]. Many of these studies have concentrated on juveniles ca. 8 months or older, bypassing the intense selection experienced during early ontogeny. However, research with GH transgenic coho salmon, *Oncorhynchus kisutch* (Walbaum), has shown phenotypic effects during early life history, including reduced survival as eyed embryos during hypoxic (low oxygen) conditions [24], advanced embryo and larval development [25]–[27] and greater susceptibility to predation and starvation as first-feeding juveniles (fry) than non-transgenic coho [28]–[30]. Collectively, these studies suggest that the relative fitness of transgenic and non-transgenic coho salmon during early life history may differ considerably in nature.

As part of a continuum of correlated traits, resting metabolism has been linked to variation in behaviour, performance, and life history strategies among individuals at both inter- and intra-specific levels [31]–[34]. In intra-specific laboratory studies with salmonids, high resting metabolic rates correlate with fast growth [35], [36], foraging-induced aggression and dominance [37]–[40]; all of which have been observed for GH transgenic salmon juveniles. Resting metabolism is the minimum energy requirement of an individual within a specific environment, and represents an internal constraint on energy allocation that has significant implications for an animal's survival [41]. For example, fish with elevated resting metabolic rates require more energy and, consequently, more oxygen to maintain essential body functions. Thus, direct and/or indirect effects of the GH transgene on resting metabolic rate during early life history may explain observations of increased sensitivity to hypoxia, advanced development, higher foraging-induced aggression and decreased anti-predator behaviour in GH transgenic salmon. However, to our knowledge, respiratory metabolism has not been compared between GH transgenic and non-transgenic salmon during early ontogeny. Moreover, previous work with GH transgenic coho salmon may not be representative of the early phenotypic responses of GH transgenic Atlantic salmon that carry a distinctly different transgene construct [42] (described in Methods).

If the GH transgene elevates metabolic rate during early ontogeny (embryo, larval and fry stages) in Atlantic salmon, as observed for older juveniles (aged >8 months) [18], [19], [23], then a similarly advanced development to that of coho salmon may result. Such phenotypic differences could influence the relative survival of transgenic and non-transgenic salmon during a critical life history period. To test for these potential phenotypic effects, and compare how they may differ from other manifestations of GH salmon transgenesis, this study compared the respiratory metabolism and development of GH transgenic and non-transgenic Atlantic salmon siblings during early ontogeny. Using siblings allowed us to control for maternal effects and general genetic background, and to thus quantify routine metabolism in GH transgenic and non-transgenic salmon at three early stages of ontogeny; eyed embryos, alevins (larvae) and fry (first-feeding juveniles) across multiple family replicates. By doing this, we aimed to isolate the genetic contributions of the transgene to offspring phenotype. Furthermore, we tested for differences in hatch time and, near exogenous feeding, alevin mass, length and the amount of yolk remaining within (transgenic versus non-transgenic) and among families.

## Methods

### Experimental Animals

A gene construct (opAFP-GHc2) consisting of growth hormone cDNA from Chinook salmon, *Oncorhynchus tshawytscha* (Walbaum), and an antifreeze protein gene promoter from ocean pout, *Macrozoarces americanus* L., was introduced into the genome of wild Atlantic salmon collected from the Exploits and Colinet Rivers, Newfoundland, Canada in 1989 [16]. A stable transgenic line (EO-1α transgene) resulting from these gene insertion experiments was produced at the Ocean Sciences Centre, Memorial University of Newfoundland [43]. During August 2005, wild adult Atlantic salmon were also collected from the Exploits River (48°55′N, 55°40′W), Newfoundland, Canada, during their return migration from sea and transferred to the Ocean Science Centre. The Exploits River salmon population is one of the largest in Newfoundland, with fish typically returning to breed following a single year at sea [44].

To isolate the genetic contributions of the transgene to offspring phenotype, 11 single family crosses were produced during 3–22 November 2005 between wild, non-transgenic females and captive-reared, transgenic males that were hemizygous for the GH transgene. True to Mendelian inheritance patterns, such crosses result in approximately half of the offspring inheriting the GH transgene [45]. This enabled the comparison of full siblings differing primarily in the presence or absence of the transgene (i.e. other genetic differences tending to be randomized), and allowed for the control of maternal effects and general genetic background.

All families were reared separately in Heath incubation trays under ambient water temperatures until first feeding. At which point, families were pooled into two distinct family groups at first feeding (5 families in one tank and 6 families in another, grouped by spawning date; ca. *n* = 500 per family) due to logistical constraints and reared in 1×1 m holding tanks. The fish were fed *ad libitum* with a combination of *Artemia* spp. and a salmonid starter dry feed (Corey Feed Mills, Fredericton, Canada). With the exception of the respirometry trials (see below), both temperature and photoperiod were kept at ambient conditions during holding and experimentation. Following all experiments, a tissue sample of each individual was screened for the transgene using the polymerase chain reaction (PCR) protocol described in [23]. All animals were treated in accordance with the guidelines provided by the Canadian Council on Animal Care and with the approval of Memorial University's Institutional Animal Care Committee (protocol 08-03-IF).

### Respirometry Equipment

To estimate the metabolic rate of individual embryos and fish, we measured their routine oxygen consumption [46] in one of two respirometry systems. Jobling [46] defined routine metabolic rate as the oxygen consumption (mg O_2_ g^−1^ hr^−1^) of fasted, unstressed animals experiencing minimal movement. In the case of endogenously feeding eyed embryos and alevins (larvae), we consider our measurements representative of routine metabolism.

The first respirometer was a custom glass design, used to measure the oxygen consumption of individual salmon eyed embryos. It consisted of an inner experimental chamber, where the animal was located, and an outer chamber connected to an external water bath (model 1150 S, VWR International, Mississauga, Canada) that maintained the inner chamber at 3°C. Freshwater was pumped into the bottom of the 6.75 ml inner chamber from an oxygenated glass reservoir (situated in the water bath) and returned through an exit port at the top of the chamber, with the aid of a peristaltic pump (Masterflex L/S model 77200-12, Cole-Palmer Inc., Barrington, USA) and low gas permeability tubing (Tygon Food & LFL, Cole Palmer Inc., Barrington, USA). Individual embryos were elevated above the bottom of the inner chamber on the mesh surface of a perforated, circular glass tube. The entire respirometer was suspended over a magnetic stirrer such that the stir bar, located within the glass tube, ensured water was mixing slowly and no oxygen gradients were present. Immediately prior to oxygen consumption measurements, the peristaltic pump was turned off and the inner chamber was closed with stop-cocks. The drop in oxygen concentration was then measured using a fibre-optic oxygen minisensor system (Fibox 3, PreSens GmbH, Regensburg, Germany; dissolved oxygen resolution: ±0.04 mg L^−1^ at 9.06 mg L^−1^) connected to a computer running OxyView software (version PST3_v532) and an oxygen sensitive spot attached to the inside surface of the inner chamber. The fibre-optic oxygen meter was calibrated regularly using aerated water and water from which all oxygen had been removed by the addition of sodium sulphite (0.1 g per 10 ml).

The second respirometer was a custom-built, glass, Blazka-type respirometer [47] that had an 82 ml volume. This device was used to measure the routine metabolism of individual alevins and fry. The design and operation of this respirometer was similar to that previously described in Killen et al. [48], with one exception. As with the respirometer used to measure embryo metabolism, water temperature was controlled using an outer water jacket that was attached to an external water bath. The water temperature was maintained at 4.5°C and 8.5°C for alevins and fry, respectively. A slow current was induced within the inner chamber to ensure proper mixing and prevent the formation of oxygen gradients. The current, however, was <3 cm s^–1^ and no swimming activity was required by the animals. A black cloth was draped over the respirometer to prevent disturbance and a mirror was used to monitor the activity of the fish during the oxygen measurement period.

### Respirometry Protocol

Fish used in the respirometry experiments were maintained at the experimental temperatures for a minimum of two weeks prior to measurement of oxygen consumption. These temperatures corresponded to the ambient conditions at the initiation of experimentation. Logistical constraints and the time consuming nature of the measurements limited the number of families that could be tested. For eyed embryos, the oxygen consumption of 6–7 embryos from six families (*n* = 39 in total; Mean ±SE = 0.135±0.003 g; Range  = 0.10–0.16 g) was measured at ages ranging from 385–415 degree days (a developmental index representing the sum of daily mean temperatures). These embryos were acclimated to the respirometer for 90 minutes prior to oxygen consumption measurements. Then two successive, 30 minute oxygen consumption measurements, separated by 15 minute periods where the respirometer was flushed with fresh water, were taken on each individual and averaged. All embryos within each family were measured within an 18 h period to limit potential developmental effects on metabolic rate. To simulate both the rearing and natural environments, all measurements were performed in total darkness.

For alevins, the oxygen consumption of 9–10 individuals from four families (*n* = 39 in total; Mean ±SE = 0.154±0.002 g; Range  = 0.12–0.19 g) was measured at ages ranging from 668–725 degree days. Individuals were acclimated to the respirometer for 90 minutes prior to a 60 minute oxygen consumption measurement. All individuals within each family were measured over 3 d to limit potential developmental effects on metabolic rate. As with the eyed embryos, all measurements were performed in total darkness.

Following one week of exogenous feeding, fry to be used for oxygen consumption measurements were haphazardly selected from the holding tanks and transferred into two aquaria (*n* = 20 per aquaria) housed in a temperature-controlled room. Fish from families having similar dates for the start to exogenous feeding (a reflection of fertilization date) were housed together. They were fasted for 48 h and acclimated to the respirometer for 150 minutes prior to a 30 minute measurement of oxygen consumption. Sixteen fry per aquarium were tested (*n* = 32 in total; Mean ±SE = 0.181±0.03 g; Range  = 0.13–0.27 g). All measurements were performed under low light conditions.

The duration of the acclimation periods was based on preliminary trials, ensuring that the animals were in a steady state of constant low oxygen consumption (i.e. they had recovered from any stress associated with handling). Rates of oxygen consumption (mg O_2_ g^−1^ hr^−1^) for each trial were calculated using the slope of a linear regression between water oxygen level (mg L^−1^) and time, then multiplied by the chamber volume and divided by the animal's mass. At the end of each day, blank measurements were made to ensure that background oxygen consumption was minimal, and the respirometers were cleaned with 100% ethanol. Any observed background oxygen consumption was subtracted from the experimental values. Trials where background oxygen consumption was greater than 5% of a fish's oxygen consumption were not included in the data set (n = 6; all in fry experiments).

### Development

Hatch time, and alevin yolk surface area (mm^2^), mass (g) and fork length (mm) near emergence (yolk sac absorption) were used as indices for examining the effect of the transgene on developmental rate. Approximately 100 eyed embryos were haphazardly sub-sampled from each of 8 families and placed into plastic canvas mesh baskets housed within separate incubation trays. During incubation, the ambient temperature ranged between 2–8°C, with a temperature of 4°C at hatch. Baskets were checked once daily for hatched individuals. At hatch, individuals were preserved in 95% ethanol for subsequent PCR analysis to determine the presence or absence of the transgene. For the same 8 families, 40 late stage alevins (ca. 774 degree days), that were approaching the start of exogenous feeding (yolk sac absorption), were haphazardly sub-sampled from the family-specific incubation trays, weighed and digitally photographed on a standardized mount using the Pixera Viewfinder 2.6 software application (Pixera Corp., Los Gatos, USA). Fork length (mm) and yolk surface area (mm^2^) were recorded using ImageJ 1.37v processing and analysis software (ImageJ, http://rsbweb.nih.gov/ij/index.html). Following measurements, the alevins were placed into individual microcentrifuge tubes containing 95% ethanol for subsequent PCR analysis.

### Data Analyses

Nested, two-way ANOVAs were performed to test for the effects of family origin and genotype (transgenic or non-transgenic) on the response variables of mass (mg) and oxygen consumption (mg O_2_ g^−1^ hr^−1^), where genotype was nested within family. Family and genotype were treated as fixed effects factors. For the respirometry experiments on fry, families were split into two distinct groups and placed in separate tanks, where tank was treated as a fixed effects factor.

To test for differences in hatch time between families and genotypes, a binomial logistic regression was fit, where the response variable represented the proportion of all individuals carrying the transgene. Explanatory variables including hatch day (represented by degree days) and family were treated as fixed effects factors. To test for the effects of family and genotype on yolk surface area (mm^2^), mass (g) and fork length (mm) of alevins near emergence (yolk sac absorption), nested, two-way ANOVAs were used with genotype nested within family. In cases where both family and genotype were statistically significant, the strength of association (effect size) between the explanatory variables and the response variable was estimated with omega-squared (ω^2^) in the ANOVA models.

All data were analyzed using the R statistical software application (version: R-2.15.3; http://www.r-project.org/). Statistical significance was measured at the 5% alpha level of type I error. Since the lmer function in the R lme4 package does not compute p-values for a nesting factor with random effects, family and tank were treated as fixed effects. An alternative mixed model analysis following a model selection approach with the Akaike information criterion (AIC) did not change any biological interpretations associated with the fixed effects ANOVA models reported.

## Results

### Respirometry

**Figure 1:**
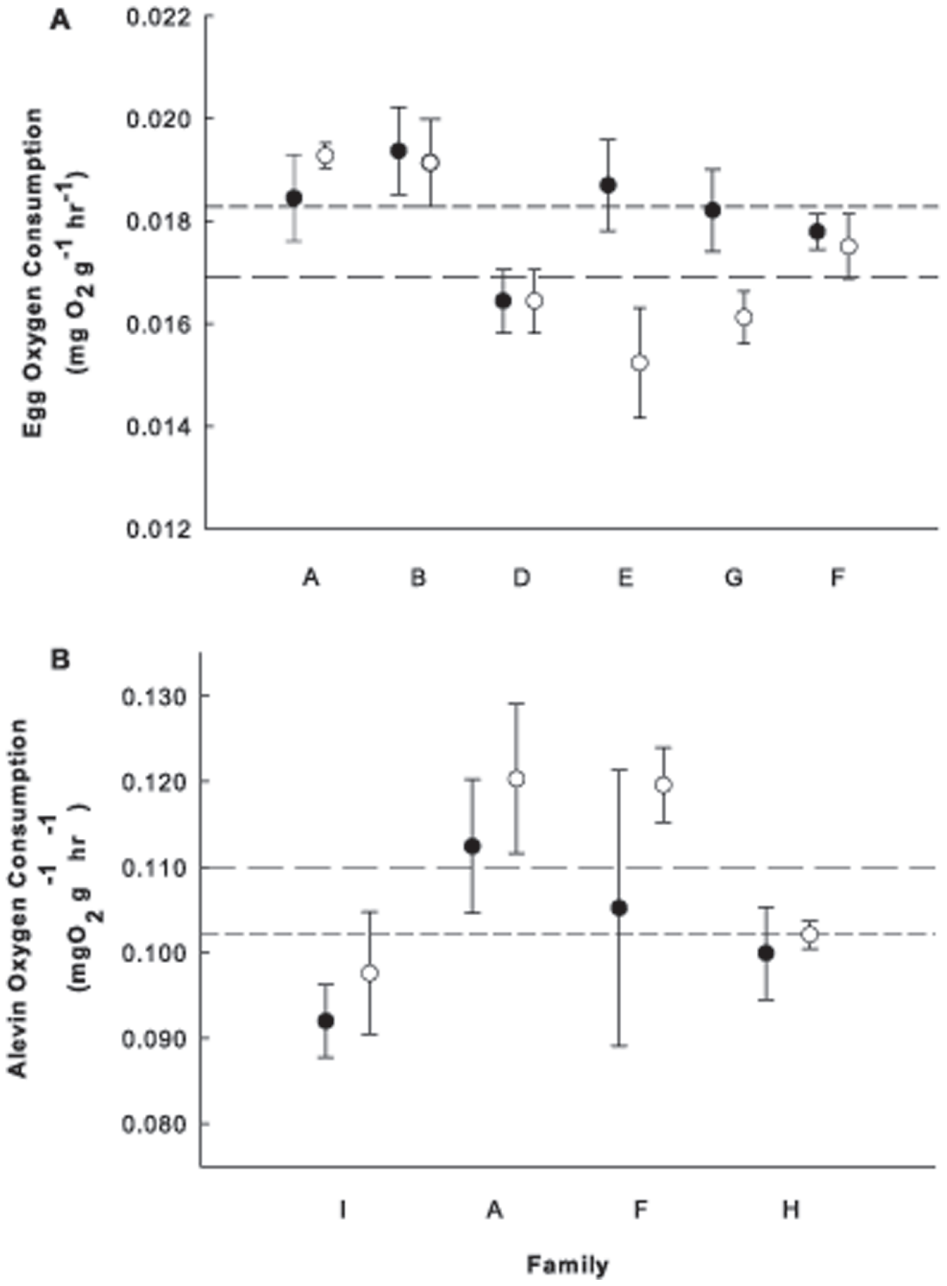
The mean (±S.E.) routine oxygen consumption (mg O_2_ g^−1^ hr^−1^) of transgenic and non-transgenic Atlantic salmon (*Salmo salar*) eyed-embryo (A) and alevin (B) full siblings. Transgenic and non-transgenic mean values within families are represented by black and white circles, respectively. The short and long dashed lines represent the overall transgenic and non-transgenic means, respectively.

At both the eyed embryo and alevin stages, oxygen consumption (MO_2_) and mass were strongly influenced by family, with less of an effect related to the transgene itself (Table 1, Fig. 1). Mean oxygen consumption of transgenic to non-transgenic siblings within families varies, as does dispersion, being higher among transgenic than non-transgenic siblings in some families and vice versa in others (Fig. 1). The overall mean oxygen consumption of transgenics was slightly higher than non-transgenics during the eyed-embryo stage, with the trend reversing at the alevin stage. However, as family effects outweighed transgene effects, the presence or absence of the transgene had little predictive value at the eyed embryo and alevin stages. Similarly, the transgene had no significant effect on oxygen consumption or mass of fry at the start of exogenous feeding (Table 1). Holding tank, reflecting fertilization date and subsequent start of exogenous feeding, however, had a significant influence on mass (First-feeding Mass: *n* = 32, F = 16.14, *P*<0.001), but not oxygen consumption (First-feeding MO_2_: *n* = 32, F = 1.1, *P* = 0.292). This likely reflects a family effect; specifically, a bias for families with larger body size in one holding tank over the other.

**Table 1:** Oxygen consumption and body mass (mean ±SE) of non-transgenic and transgenic eyed embryos, alevins (larvae) and fry (juveniles at the start of exogenous feeding) measured using respirometry.

			Transgene Effect				Family Effect	
Trait	Non-transgenic	Transgenic	N	d.f.	F	P	F	P
**Eyed Embryos**								
Oxygen Consumption (mg O_2_ g^−1^ hr^−1^)	0.017±0.000	0.018±0.000	39	11,27	1.83	0.130	4.04	0.007
Mass (g)	0.133±0.004	0.137±0.004	39	11,27	1.26	0.310	64.85	<0.001
**Alevins**								
Oxygen Consumption (mg O_2_ g^−1^ hr^−1^)	0.110±0.003	0.102±0.004	39	7,31	0.75	0.567	4.91	0.007
Mass (g)	0.154±0.003	0.154±0.004	39	7,31	0.36	0.832	36.22	<0.001
**Fry**								
Oxygen Consumption (mg O_2_ g^−1^ hr^−1^)	0.164±0.007	0.170±0.004	32	3,29	1.07	0.310	—	—
Mass (g)	0.172±0.010	0.187±0.007	32	3,29	0.62	0.340	—	—

ANOVA results for the two categorical effects of transgene and family are also presented. At the start of feeding, different family groups were pooled into two tanks for logistical reasons and thus there is no measure of family effects for fry.

### Development

**Figure 2:**
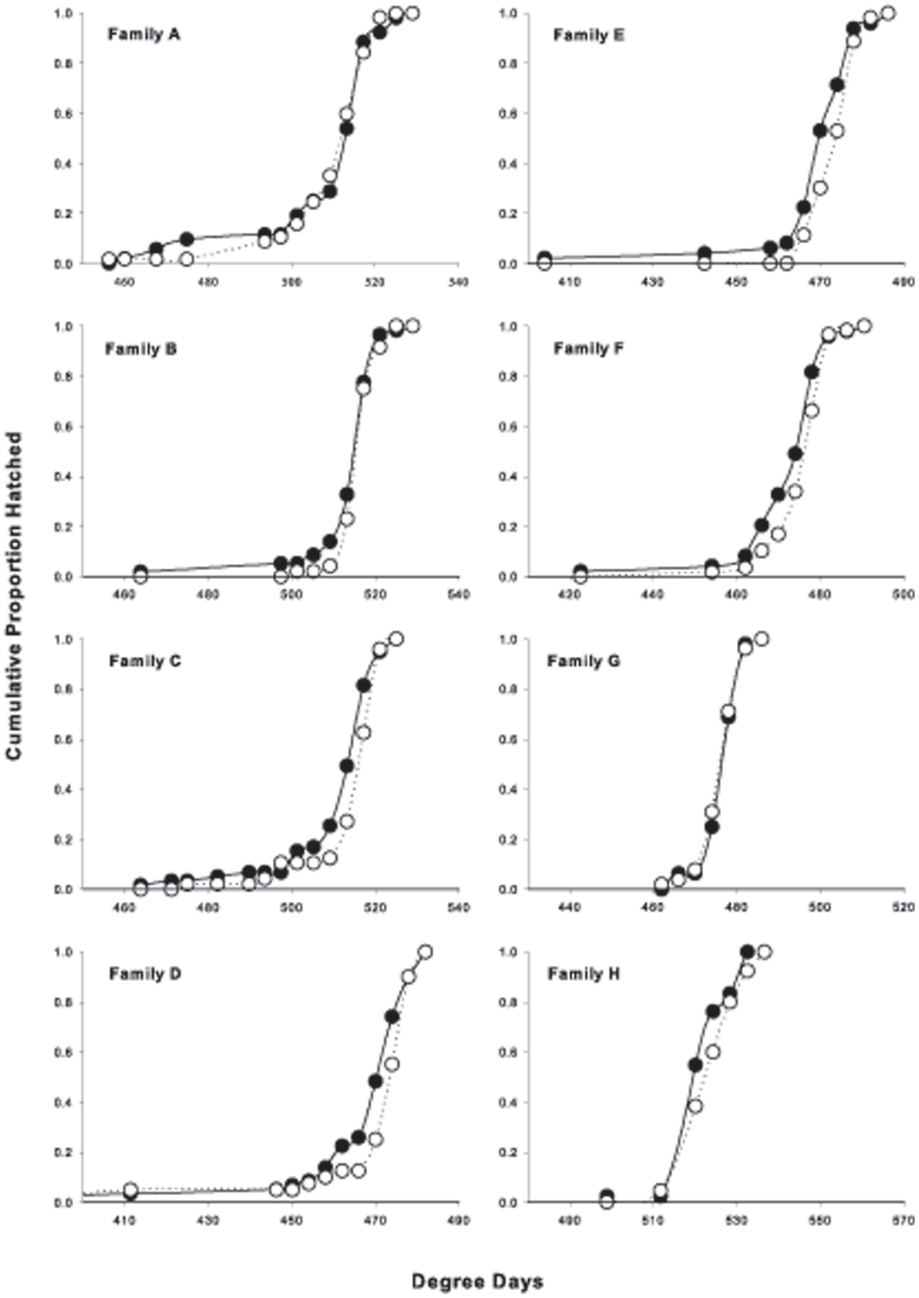
The time of hatch (degree days) of full-sibling transgenic and non-transgenic Atlantic salmon (*Salmo salar*) from eight families. Transgenic and non-transgenic values are represented by black and white circles, respectively. These data are represented by cumulative proportions of approximately 100 individuals per family.

The majority of individuals (>60%) within each family hatched over a three to four day period (Fig. 2). The proportion of transgenics hatched was influenced by both family (*n* = 837, χ^2^ = 20.48, *P* = 0.004) and accumulated degree days (*n* = 837, χ^2^ = 13.00, *P*<0.001). Transgenic individuals tended to hatch less than one day (i.e. 4 degree days) earlier (Transgenic mean ±SE = 493.8±8.2 degree days, Non-transgenic 497.2±8.1 degree days). When comparing the time to hatch of transgenic and non-transgenic individuals within families, it appears that the tendency for transgenics to hatch earlier was strong in some families (e.g. E, F; Fig. 2) and weak in others (e.g. A, G). Thus, the effect of transgenesis on hatch time was to some extent dependent on family.

**Figure 3:**
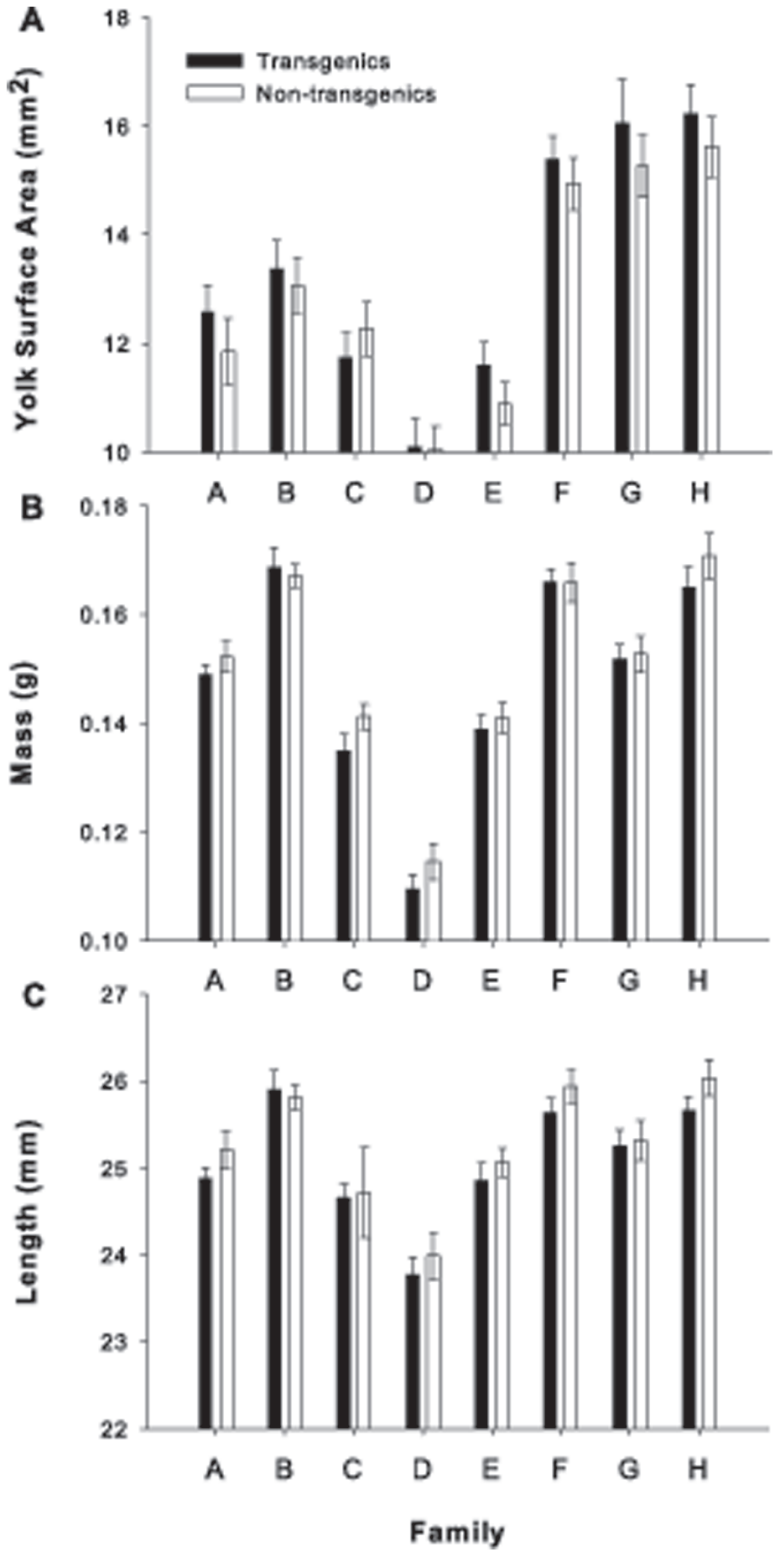
Mean (±S.E.) yolk surface area (A; mm^2^), mass (B; g), and fork length (C; mm) of transgenic and non-transgenic Atlantic salmon (*Salmo salar*) alevins near emergence.

Near emergence (i.e. yolk sac nearly absorbed and prior to exogenous feeding), transgenic alevins had a slightly (3%) greater amount of yolk remaining than non-transgenics (Table 2). Family of origin, however, significantly influenced the amount of yolk sac remaining and had a substantially larger effect size than that of the transgene itself (Table 2; Fig. 3). The size, mass and length, of the transgenics at this stage tended to be slightly smaller than that of the non-transgenics (Table 2; Fig. 3). As with the amount of yolk sac remaining, however, family effects demonstrated a greater influence on alevin size than did the presence of the transgene (Table 2; Fig. 3). Overall, there was greater variation in body size among families than between transgenic and non-transgenic alevins within families.

**Table 2:** Amount of yolk sac remaining and size (mean ±SE) of non-transgenic and transgenic alevins (larvae) near the start of exogenous feeding.

			Transgene Effect					Family Effect		
Trait	Non-transgenic	Transgenic	N	d.f.	F	P	ω^2^	F	P	ω^2^
Yolk sac remaining (mm^2^)	12.990±0.260	13.380±0.270	316	15,300	2.21	0.027	0.008	132.58	<0.001	0.739
Body mass (g)	0.151±0.002	0.148±0.000	316	15,300	3.10	0.022	0.006	318.30	<0.001	0.800
Body length (mm)	25.260±0.120	25.080±0.090	316	15,300	1.66	0.107	—	54.77	<0.001	—

ANOVA results, including relevant effect sizes as estimated by omega-squared (ω^2^) values, are shown for the two categorical effects of transgene and family.

## Discussion

Family differences had a stronger influence on the routine oxygen consumption (metabolism) and developmental rate of Atlantic salmon during early ontogeny than did GH transgenesis (Fig. 1–3). Transgenesis did not affect the oxygen consumption of individuals at the eyed-embryo, alevin (larval) or fry (first feeding juvenile) stages in a consistent way among families. The majority of individuals (>60%) within each family hatched over a three or four day period and the effect of transgenesis was weak. Transgenic fish hatched less than one day earlier than their non-transgenic siblings. Conversely, near emergence, transgenic individuals contained more yolk and were smaller in terms of both mass and length. However, the influence of genotype on all these measures was less than that of family, suggesting that family of origin contributes more to the variation of these traits than the GH transgene.

The vulnerability of salmonid embryos to low oxygen conditions has been demonstrated previously [9], [49], [50]. If GH transgenesis were to affect the basal metabolic rate of Atlantic salmon embryos, there could be survival differences during embryo incubation relative to non-transgenic individuals [24], [51]. Both metabolic and developmental measurements, however, were similar between transgenic and non-transgenic embryos of Atlantic salmon, suggesting that the threat of exposure to periods of hypoxia in the gravel beds would be similar.

Like many other fishes, the transition from endogenous to exogenous feeding is considered a critical period of survival for stream-dwelling salmonids [13]–[15]. Suitable spawning habitat can contain dense aggregations of nests, a situation that results in density-dependent competition among emerging fry for foraging territories [12], [52]. Body size at emergence and timing of emergence are thought to be important determinants of survival during this period. Larger fish tend to win laboratory-based contests against smaller fish, and this has been shown to carry over to the performance of individuals in wild release experiments [14], [53], [54]. However, the advantages and disadvantages of emerging early or late, relative to the rest of the population, are likely dependent on local environmental conditions. Early emergence may provide a beneficial opportunity to establish prime foraging territories (prior residency), and perhaps, an additional chance to grow [55]–[58]. Conversely, environmental stressors such as temporal variation in predation pressure, food resources and suitable habitat characteristics provide possible selective pressures against early emergence [11], [59], [60]. Thus, transgene-induced changes in body size at emergence and/or the timing of emergence, in either direction, have the potential to influence the fitness of the transgene in nature.

While consistent differences were observed in yolk area, mass and length between transgenic and non-transgenic alevins close to emergence (start of exogenous feeding), the extent to which such small differences affect relative fitness at emergence is unclear. It was evident, however, that family of origin was responsible for more variation in alevin characteristics than was the transgene. From a population perspective, the most dominant trait influencing emergence time may be spawning time [61]–[63]. If we are to assume that the transgene does not influence female spawn time, then the key traits influencing fitness at emergence are the rate of development (emergence time) and size at emergence. In the current study, non-transgenic alevins contained less yolk reserves and were slightly larger near emergence, suggesting transgenic Atlantic salmon may be more susceptible to predation (cf. [64]) and competitively disadvantaged at the onset of first-feeding. However, the differences in the mean value of these measurements between transgenic and non-transgenic individuals were 3% or less (Fig. 3). Salmonid fry have demonstrated considerable variation in the amount of yolk remaining at emergence [65]–[67]. Thus, the small differences observed in yolk reserves between transgenics and non-transgenics suggest that emergence time would be similar, unless the transgene affects emergence behaviour. Previous studies assessing the effect of emergence time on performance in the wild have found that early emergence provides a competitive advantage. However, such studies have either compared individuals with substantial differences in emergence time (5–6 days) [14] or the early emerging group had a confounding, albeit natural, size advantage [15]. The body size differences detected in the current study may be so small (often <1 mm) as to not influence contests for foraging territories in stream salmonids as suggested by previous behavioural experiments [30], [68], [69]. Thus, the high levels of family variation combined with the small transgene–induced differences in characteristics of alevin siblings near emergence suggest that transgenesis may not have a considerable influence on fitness related to body size at, and timing of first-feeding.

The similarity in metabolic and developmental rate measures of GH transgenic and non-transgenic Atlantic salmon siblings contrasts with observations made with GH transgenic coho salmon during early ontogeny. GH transgenic coho salmon have been shown to experience increased mortality under hypoxic conditions [24], hatch 2–3 days earlier [25], [27] and emerge from the gravel 1–2 weeks earlier [26], [27]. An increased sensitivity to hypoxic conditions suggests higher basal metabolic rates [35], as observed in older GH transgenic salmonids [19], [23]. A higher metabolic rate during early ontogeny may speed up the mobilization of yolk-sac reserves to body tissues and/or for maintenance processes [35], and is thus, a plausible explanation for observations of advanced development to first-feeding and greater susceptibility of eyed embryos to low oxygen conditions in GH transgenic coho salmon carrying the OnMTGHI gene construct. However, the current study has shown the opAFP-GHc2 gene construct (EO-1α line) has little to no consistent phenotypic effect on pre-emergent Atlantic salmon. This suggests that there are ecologically important phenotypic differences between these two GH transgenic lines during this critical period of survival. Whether this is due to the different growth hormone constructs or different species effects is not known.

Elevated metabolic rate has been shown to correlate with fast growth [35], [36], foraging-induced aggression and dominance [37]–[40]. In addition, it is hypothesized that higher basal metabolic rates concomitantly increase energy requirements that are addressed by a suite of compensatory behavioural changes toward greater foraging motivation and risk taking actions [34], [70], [71]. GH transgenic coho salmon juveniles, from as young as the fry stage, have shown changes in behaviour and performance that are consistent with this hypothesis [28], [29]. The current study is the first to measure the respiratory metabolism of GH transgenic fish at first-feeding, and we find no consistent effect of transgenesis on the metabolic rate of salmon fry up to one month following emergence. Our results support the findings of a study conducted concurrently, where Moreau et al. [30] observed no differences in the competitive ability or survival of first-feeding GH transgenic and non-transgenic Atlantic salmon fry reared in low feed, near-natural stream environments. Previous work has indicated mRNA expression of the transgene, GH1 and GH2 receptors as early as the embryo stage (King and Fletcher, Unpub. Data, Young and Fletcher Unpub. Data). Therefore, collectively, our work indicates that there is a delay in the response of other physiological systems to elevated levels of growth hormone and suggests that fitness may not be greatly affected during this critical period of early ontogeny. Previous measurements on older GH transgenic Atlantic salmon juveniles (>2 months post-emergence) have demonstrated elevated routine metabolic rates that are consistent with shifts in behaviour and performance relative to non-transgenics [18], [72]. We have observed changes in growth prior to this stage of development (personal observations); however, the absence of an effect during this most critical period of survival suggests that the early recruitment of transgenic juveniles may be similar to that of non-transgenic individuals in the wild.

## Conclusions

In the current study, we controlled for genetic background by comparing transgenic and non-transgenic full siblings. This approach was intended to not only isolate the effects of the transgene from differences in genetic background, but also to minimize maternal effects that may confound interpretation of direct genetic effects. We found that family of origin explained considerably more trait variation than did transgenesis. Pakkasmaa et al. [73] found a similarly strong family effect on the metabolic rate of Arctic charr (*Salvelinus alpinus*) eyed embryos. This suggests that any potential selection acting upon the GH transgene during early life history may be overshadowed by selection acting at the family level. This finding is relevant to understanding the potential implications of the offspring of GH transgenic fish escapees, particularly as it concerns Atlantic salmon. Firstly, the fitness of transgenic offspring in early life may be more a function of background genotype (i.e. due to non-local origins and domestication selection) than transgenesis itself. Secondly, the extent and form of differences between transgenic and non-transgenic siblings within families varied in the present study, and as such, this could be indicative of the transgene having different effects in different genetic backgrounds (i.e. epistasis) [74]. Thus, the strong effect of family (i.e. background genotype) contributes to the complexity and adds to the uncertainty [6], [75] of predicting the fate of the transgene in nature.

## References

[1] Ferguson A, Fleming IA, Hindar K, Skaala Ø, McGinnity P, et al. (2007) Farm Escapes. In: Verspoor E, Stradmeyer L, Nielsen J, editors. Atlantic Salmon: Genetics, Conservation and Management.Oxford: Blackwell Publishing. pp. 367–409.

[2] Morris MRJ, Fraser DJ, Heggelin AJ, Whoriskey FG, Carr JW, et al (2008) Prevalence and recurrence of escaped farmed Atlantic salmon (*Salmo salar*) in eastern North American rivers. Can J Fish Aquat Sci 65: 2807–2826.

[3] Thorstad EB, Fleming IA, McGinnity P, Soto D, Wennevik V, et al (2008) Incidence and impacts of escaped farmed Atlantic salmon (Salmo salar) in nature. NINA Special Report 36: 110pp.

[4] Kapuscinski AR, Hallerman EM (1991) Implications of introduction of transgenic fish into natural ecosystems. Can J Fish Aquat Sci 48: 99–107.

[5] Muir WM, Howard RD (2002) Assessment of possible ecological risks and hazards of transgenic fish with implications for other sexually reproducing organisms. Transgenic Res 11: 101–114. 12054344 10.1023/a:1015203812200

[6] Devlin RH, Sundström LF, Muir WM (2006) Interface of biotechnology and ecology for environmental risk assessments of transgenic fish. Trends Biotechnol 24: 89–97. 16380181 10.1016/j.tibtech.2005.12.008

[7] Lacroix GL (1985) Survival of eggs and alevins of Atlantic salmon (Salmo-salar) in relation to the chemistry of interstitial water in redds in some acidic streams of Atlantic Canada. Can J Fish Aquat Sci 42: 292–299.

[8] Chapman DW (1988) Critical-review of variables used to define effects of fines in redds of large salmonids. Trans Am Fish Soc 117: 1–21.

[9] Peterson NP, Quinn TP (1996) Spatial and temporal variation in dissolved oxygen in natural egg pockets of chum salmon, in Kennedy Creek, Washington. J Fish Biol 48: 131–143.

[10] Chandler GL, Bjornn TC (1988) Abundance, growth, and interactions of juvenile steelhead relative to time of emergence. Trans Am Fish Soc 117: 432–443.

[11] Brännäs E (1995) First access to territorial space and exposure to strong predation pressure: A conflict in early emerging Atlantic salmon (Salmo salar L.) fry. Evol Ecol 9: 411–420.

[12] Einum S, Nislow KH (2005) Local-scale density-dependent survival of mobile organisms in continuous habitats: an experimental test using Atlantic salmon. Oecologia 143: 203–210. 15654640 10.1007/s00442-004-1793-y

[13] Elliott JM (1994) Quantitative ecology and the brown trout. Oxford: Oxford University Press.

[14] Einum S, Fleming IA (2000) Selection against late emergence and small offspring in Atlantic salmon (*Salmo salar*). Evolution 54: 628–639. 10937238 10.1111/j.0014-3820.2000.tb00064.x

[15] Nislow KH, Einum S, Folt CL (2004) Testing predictions of the critical period for survival concept using experiments with stocked Atlantic salmon. J Fish Biol 65: 188–200.

[16] Du SJ, Gong ZY, Fletcher GL, Shears MA, King MJ, et al (1992) Growth enhancement in transgenic Atlantic salmon by the use of an all fish chimeric growth-hormone gene construct. Biotechnology 10: 176–181. 1368229 10.1038/nbt0292-176

[17] Devlin RH, Yesaki TY, Biagi CA, Donaldson EM, Swanson P, et al (1994) Extraordinary salmon growth. Nature 371: 209–210.

[18] Abrahams MV, Sutterlin AM (1999) The foraging and antipredator behaviour of growth-enhanced transgenic Atlantic salmon. Anim Behav 58: 933–952. 10564595 10.1006/anbe.1999.1229

[19] Cook JT, McNiven MA, Sutterlin AM (2000) Metabolic rate of pre-smolt growth-enhanced transgenic Atlantic salmon (Salmo salar). Aquaculture 188: 33–45.

[20] Leggatt RA, Devlin RH, Farrell AP, Randall DJ (2003) Oxygen uptake of growth hormone transgenic coho salmon during starvation and feeding. J Fish Biol 62: 1053–1066.

[21] Sundström FL, Devlin RH, Johnsson JI, Biagi CA (2003) Vertical position reflects increased feeding motivation in growth hormone transgenic coho salmon (*Oncorhynchus kisutch*). Ethology 109: 701–712.

[22] Tymchuk WEV, Abrahams MV, Devlin RH (2005) Competitive ability and mortality of growth-enhanced transgenic coho salmon fry and parr when foraging for food. Trans Am Fish Soc 134: 381–389.

[23] Deitch EJ, Fletcher GL, Petersen LH, Costa IASF, Shears MA, et al (2006) Cardiorespiratory modifications, and limitations, in post-smolt growth hormone transgenic Atlantic salmon Salmo salar. J Exp Biol 209: 1310–1325. 16547302 10.1242/jeb.02105

[24] Sundt-Hansen L, Sundström LF, Einum S, Hindar K, Fleming IA, et al (2007) Genetically enhanced growth causes increased mortality in hypoxic environments. Biol Lett 3: 165–168. 17272234 10.1098/rsbl.2006.0598 PMC2375932

[25] Devlin RH, Biagi CA, Yesaki TY (2004) Growth, viability and genetic characteristics of GH transgenic coho salmon strains. Aquaculture 236: 607–632.

[26] Sundström LF, Löhmus M, Devlin RH (2005) Selection on increased intrinsic growth rates in coho salmon, *Oncorhynchus kisutch* . Evolution 59: 1560–1569. 16153041

[27] Löhmus M, Sundström LF, Björklund M, Devlin RH (2010) Genotype-temperature interaction in the regulation of development, growth, and morphometrics in wild-type, and growth-hormone transgenic coho salmon. PLoS ONE 5: 1–11. 10.1371/journal.pone.0009980 PMC2848618 20376315

[28] Devlin RH, D'Andrade M, Uh M, Biagi CA (2004) Population effects of growth hormone transgenic coho salmon depend on food availability and genotype by environment interactions. Proc Nat Acad Sci 101: 9303–9308. 15192145 10.1073/pnas.0400023101 PMC438972

[29] Sundström LF, Löhmus M, Johnsson JI, Devlin RH (2004) Growth hormone transgenic salmon pay for growth potential with increased predation mortality. Proc Roy Soc Lond, B 271: S350–S352. 10.1098/rsbl.2004.0189 PMC1810071 15504015

[30] Moreau DTR, Fleming IA, Fletcher GL, Brown JA (2011) Growth hormone transgenesis does not influence territorial dominance or growth and survival of first-feeding Atlantic salmon *Salmo salar* L. in food-limited stream microcosms. J Fish Biol 78: 726–740. 21366569 10.1111/j.1095-8649.2010.02888.x

[31] Symonds MRE (1999) Life histories of the Insectivora: the role of phylogeny, metabolism and sex differences. J Zool 249: 315–377.

[32] Sih A, Bell AM, Johnson JC, Ziemba RE (2004) Behavioural syndromes: an integrative overview. Quart Rev Biol 79: 241–277. 15529965 10.1086/422893

[33] Biro PA, Stamps JA (2008) Are animal personality traits linked to life-history productivity? Trends Ecol Evol 23: 361–368. 18501468 10.1016/j.tree.2008.04.003

[34] Careau V, Thomas D, Humphries MM, Reale D (2008) Energy metabolism and animal personality. Oikos 117: 641–653.

[35] Metcalfe NB, Taylor AC, Thorpe JE (1995) Metabolic rate, social status and life-history strategies in Atlantic salmon. Anim Behav 49: 431–436.

[36] Yamamoto T, Ueda H, Higashi S (1998) Correlation among dominance status, metabolic rate and otolith size in masu salmon. J Fish Biol 52: 281–290.

[37] Cutts CJ, Metcalfe NB, Taylor AC (1998) Aggression and growth depression in juvenile Atlantic salmon: the consequences of individual variation in standard metabolic rate. J Fish Biol 52: 1026–1037.

[38] Cutts CJ, Adams CE, Campbell A (2001) Stability of physiological and behavioural determinants of performance in Arctic char (Salvelinus alpinus). Can J Fish Aquat Sci 58: 961–968.

[39] McCarthy ID (2001) Competitive ability is related to metabolic asymmetry in juvenile rainbow trout. J Fish Biol 59: 1002–1014.

[40] Lahti K, Huuskonen H, Laurila A, Piironen J (2002) Metabolic rate and aggressiveness between brown trout populations. Funct Ecol 16: 167–174.

[41] Brown JH, Gillooly JF, Allen AP, Savage VM, West GB (2004) Toward a metabolic theory of ecology. Ecology 85: 1771–1789.

[42] Nam YK, Maclean N, Fu C, Pandian TJ, Eguia MRR (2007) Development of transgenic fish: scientific background. In: Kapuscinski AR, Hayes KR, Li S, Dana G, editors. Environmental risk assessment of genetically modified organisms: methodologies for transgenic fish. Cambridge: CAB International.pp. 61–94.

[43] Yaskowiak ES, Shears MA, Agarwal-Mawal A, Fletcher GL (2006) Characterization and multi-generational stability of the growth hormone transgene (EO-1 alpha) responsible for enhanced growth rates in Atlantic salmon. Transgenic Res 15: 465–480. 16906447 10.1007/s11248-006-0020-5

[44] O'Connell MF, Dempson JB, Mullins CC, Reddin DG, Bourgeois CE, et al (2003) Status of Atlantic salmon (*Salmo salar* L.) stocks of insular Newfoundland (SFAs 3–14A). Canadian Scientific Advisory Secretariat Research Document 2003/002, 1–60.

[45] Shears MA, King MJ, Goddard SV, Fletcher GL (1992) Gene transfer in salmonids by injection through the micropyle. IN: Hew CL, Fletcher GL, editors. Transgenic Fish.Singapore: World Scientific. pp. 44–60.

[46] Jobling M (1994) Respiration and Metabolism. In: Jobling M, editor. Fish Bioenergetics London: Chapman & Hall. pp. 61–94.

[47] Brett JR (1964) The respiratory metabolism and swimming performance of young sockeye salmon. J Fish Res Board Can 21: 1183–1226.

[48] Killen SS, Gamperl AK, Brown JA (2007) Ontogeny of predator-sensitive foraging and routine metabolism in larval shorthorn sculpin, Myoxocephalus scorpius. Mar Biol 152: 1249–1261.

[49] Rubin JF, Glimsater C (1996) Egg-to-fry survival of the sea trout in some streams of Gotland. J Fish Biol 48: 585–606.

[50] Einum S, Hendry AP, Fleming IA (2002) Egg-size evolution in aquatic environments: does oxygen availability constrain size? Proc Roy Soc Lond, B 269: 2325–2330. 10.1098/rspb.2002.2150 PMC1691158 12495499

[51] Alderdice DF, Wickett WP, Brett JR (1958) Some effects of temporary exposure to low dissolved oxygen levels on Pacific salmon eggs. J Fish Res Board Can 15: 229–249.

[52] Einum S, Nislow KH, McKelvey S, Armstrong JD (2008) Nest distribution shaping within-stream variation in Atlantic salmon juvenile abundance and competition over small spatial scales. J Anim Ecol 77: 167–172. 18005129 10.1111/j.1365-2656.2007.01326.x

[53] Johnsson JI (1993) Big and brave: size selection affects foraging under risk of predation in juvenile rainbow trout, Oncorhynchus mykiss. Anim Behav 45: 1219–1225.

[54] Rhodes JS, Quinn TP (1998) Factors affecting the outcome of territorial contests between hatchery and naturally reared coho salmon parr in the laboratory. J Fish Biol 53: 1220–1230.

[55] Cutts CJ, Brembs B, Metcalfe NB, Taylor AC (1999) Prior residence, territory quality and life-history strategies in juvenile Atlantic salmon (Salmo salar L.). J Fish Biol 55: 784–794.

[56] O'Connor KI, Metcalfe NB, Taylor AC (2000) The effects of prior residence on behaviour and growth rates in juvenile Atlantic salmon (*Salmo salar*). Behav Ecol 11: 13–18.

[57] Johnsson JI, Forser A (2002) Residence duration influences the outcome of territorial conflicts in brown trout (Salmo trutta). Behav Ecol Sociobiol 51: 282–286.

[58] Skoglund H, Einum S, Robertson G (2011) Competitive interactions shape offspring performance in relation to seasonal timing of emergence in Atlantic salmon. J Anim Ecol 80: 365–374. 21155770 10.1111/j.1365-2656.2010.01783.x

[59] Jensen AJ, Johnsen BO (1999) The functional relationship between peak spring floods and survival and growth of juvenile Atlantic salmon (Salmo salar) and brown trout (Salmo trutta). Funct Ecol 13: 778–785.

[60] Nislow KH, Folt CL, Parrish DL (2000) Spatially explicit bioenergetic analysis of habitat quality for age-0 Atlantic salmon. Trans Am Fish Soc 129: 1067–1081.

[61] Brannon EL (1987) Mechanisms stabilizing salmonid fry emergence timing. Canadian Special Publication of Fisheries and Aquatic Sciences, 96: , 120–124.

[62] Heggberget TG (1988) Timing of spawning in Norwegian Atlantic salmon (Salmo-salar). Can J Fish Aquat Sci 45: 845–849.

[63] Fleming IA (1996) Reproductive strategies of Atlantic salmon: ecology and evolution. Rev Fish Biol Fish 6: 379–416.

[64] Rollinson N, Hutchings JA (2010) Why does egg size increase with maternal size? Effects of egg size and egg density on offspring phenotypes in Atlantic salmon (*Salmo salar*). Evol Ecol Res 12: 949–960.

[65] Garcia de Leaniz C, Fraser N, Huntingford FA (2000) Variability in performance in wild Atlantic salmon, Salmo salar L., fry from a single redd. Fish Manag Ecol 7: 489–502.

[66] Skoglund H, Barlaup BT (2006) Feeding pattern and diet of first feeding brown trout fry under natural conditions. J Fish Biol 68: 507–521.

[67] Rollinson N, Hutchings JA (2011) Why does egg size of salmonids increase with the mean size of population spawning gravels? Can J Fish Aquat Sci 68: 1307–1315.

[68] Johnsson JI, Petersson E, Jönsson E, Järvi T, Björnsson BT (1999) Growth hormone-induced effects on mortality, energy status and growth: a field study on brown trout (Salmo trutta). Funct Ecol 13: 514–522.

[69] Metcalfe NB, Valdimarsson SK, Morgan IJ (2003) The relative roles of domestication, rearing environment, prior residence and body size in deciding territorial contests between hatchery and wild juvenile salmon. J Appl Ecol 40: 535–544.

[70] Cutts CJ, Metcalfe NB, Taylor AC (2002) Juvenile Atlantic Salmon (Salmo salar) with relatively high standard metabolic rates have small metabolic scopes. Funct Ecol 16: 73–78.

[71] Biro PA, Abrahams MV, Post JR, Parkinson EA (2006) Behavioural trade-offs between growth and mortality explain evolution of submaximal growth rates. J Anim Ecol 75: 1165–1171. 16922852 10.1111/j.1365-2656.2006.01137.x

[72] Stevens ED, Sutterlin A, Cook T (1998) Respiratory metabolism and swimming performance in growth hormone transgenic Atlantic salmon. Can J Fish Aquat Sci 55: 2028–2035.

[73] Pakkasmaa S, Penttinen OP, Piironen J (2006) Metabolic rate of Arctic charr eggs depends on their parentage. J Comp Physiol B, Biochem Syst Environ Physio 176: 387–391. 10.1007/s00360-005-0057-4 16362308

[74] Lehner B (2011) Molecular mechanisms of epistasis within and between genes. Trends Genet 27: 323–331. 21684621 10.1016/j.tig.2011.05.007

[75] Moreau DTR (2014) Ecological risk analysis and genetically modified salmon: Management in the face of uncertainty. Annu. Rev. Anim. Biosci 2: 515–533. 25384154 10.1146/annurev-animal-022513-114231

